# Factors that influence full-time MPH Students’ willingness in China: would You apply again for an MPH graduate degree if you had another opportunity?

**DOI:** 10.1186/s12909-017-0873-8

**Published:** 2017-02-14

**Authors:** Nan Wang, Jinzhong Jia, Ke Wu, Yuanyuan Wang, Chi Zhang, Wei Cao, Liping Duan, Zhifeng Wang

**Affiliations:** 1 School of Public Health, Peking University, Beijing, China; 2 Health Science Center, Peking University Graduate School, Beijing, China; 3 Beijing Hospital, Beijing, China

**Keywords:** MPH, Satisfaction, Attitudes, Factors

## Abstract

**Background:**

Current and emerging challenges to public health in the 21st century are vastly different from those faced in previous centuries. And the shortage of health personnel and their low level of educational qualifications hindered the development of Chinese public health services. In order to fulfill this requirement, the Ministry of Education initiated a full-time, Master of Public Health (MPH) graduate programme in 2009. This study aimed to evaluate the level of graduate students’ satisfaction with full-time Master of Public Health (MPH) education in China, and whether they would apply again for an MPH graduate degree if they had another opportunity to do so, as well as to identify the factors influencing their decision-making process.

**Methods:**

An anonymous, web-based survey questionnaire containing 61 items was distributed to 702 MPH students in 35 universities or colleges. The questions covered the categories of student admission, training goals, lecture courses, practical training, research activities and mentorship. Levels of satisfaction were compared between MPH students who would choose MPH again as their graduate degree if they had another opportunity to do so and those who would not. Key influencing factors of training satisfaction were identified using logistic regression models.

**Results:**

A total of 65.10% of the participants would apply again for MPH education if they had another opportunity to do so. The factors influencing students’ willingness included their university type, the time since admission and their initial willingness. In addition, the four common factors (admissions & lecture courses, research activities & mentorship, practical training and training goals) emerging from factor analysis were all significantly positively correlated with student willingness (*p* < 0.001).

**Conclusions:**

Most MPH students surveyed were highly satisfied with their MPH education and, although they advocated for improvements and reforms in some aspects, they would still choose MPH as their graduate degree again if they had another opportunity to do so.

## Background

Current and emerging challenges to public health in the 21st century are vastly different from those faced in previous centuries [1]. With China’s economic and social development, an increasing variety of public health issues are a threat [2, 3], and have resulted in serious challenges for public health professionals. Up until 2014, the total number of professional staff in Chinese public health institutions was only 87.5 million, which was far below the target of 95 million set for 2015 [4]. Moreover, the majority of health professionals held tertiary qualifications; only 4.2% of public health practitioners and practicing physicians had a graduate degree [4]. The shortage of health personnel and their low level of educational qualifications hindered the development of Chinese public health services. Therefore, a strengthening of training initiatives and the cultivation of high-level, application-oriented public health personnel was needed. In order to fulfill this requirement, the Ministry of Education initiated a full-time, Master of Public Health (MPH) graduate programme in 2009, and this marked a major change in China’s public health education from academy-orientated to an application-centered model [5]. As of 2013, China had 44 universities or colleges that could recruit full-time MPH students. Full-time MPH education is unique in China, because it has been developed from the academic degree of public health to cultivate high-level public health talents. Since there is no formal assessment of the educational quality of the full-time MPH programme, universities and colleges have an urgent need for scientific evidence in this regard. To date, we have found scarcely any studies on this topic, via online database searches, either in Chinese or in other languages [6–8]. This existing knowledge gap poses a considerable barrier to the reform and development of MPH education in China.

*The Medium- and Long-term Plan on Medical Personnel Development (from 2011 to 2020)* issued by the Ministry of Health (now known as the National Health and Family Planning Commission) of the People’s Republic of China in 2011 indicated the improvement of education quality as a core mission of medical education reform and development [9]. It is generally agreed that any education programme must be evaluated for quality assurance and further improvement [10]. However, as a major component of China’s education system, the evaluation of graduate students and their mentors has often been ignored [11]. According to Kirkpatrick’s evaluation model, four criteria, namely, reaction, learning, behaviour and results, can be used to assess a training programme. Reaction indicates the participants’ thinking and feelings towards the training (satisfaction); learning measures the extent to which principles, facts and techniques have been understood and absorbed; behaviour aims to evaluate how well the students can use the knowledge, skills, and/or attitudes learnt from classrooms in their jobs; and results measure the ends, goals and performance [12, 13]. Reaction is the foundation of the evaluation model, on the basis of which one can learn the flaws of an educational programme and make improvements [10, 13].

Today, a greater number of universities and colleges worldwide have realised the importance of evaluating students’ satisfaction with their educational experiences, and have been attempting to obtain useful, relevant feedback in a variety of ways [14–17]. In the United States, the Student Satisfaction Inventory™ had reached over 600,000 college students by the end of 2014. The United Kingdom also conducted a students’ experience survey, which covered aspects including campus life, service facilities, lecture courses planning, teaching, practice, registration and guidance, as an important part of the new higher education quality assurance system, with a focus on interactions between students and schools [18]. Some Canadian authors used core competencies to build an evaluative framework for the MPH degree programme, primarily concerning the curriculum and course structure, practical placement, faculty and programme experience [19]. In comparison with these developed countries, the Chinese students’ satisfaction survey system still lags behind the development of higher education; there are a limited number of articles on the satisfaction level of undergraduate students [20, 21], and even fewer studies have been conducted on graduate students, irrespective of those in the public health speciality. In addition, we should be aware that, for students, it’s difficult to evaluate the MPH programme by assessing how they apply, in practice, the knowledge and skills they have acquired from that programme. Therefore, MPH students’ willingness to choose their speciality again, if they had another chance to do so, is a good reflection of the quality of the programme.

In some countries, the MPH programme is integrated with medical degree studies [22]. One qualitative study conducted in Iran found that factors, including the acquisition of health-related knowledge, gaining a perspective beyond clinical practice, obtaining a degree to strengthen an academic résumé, immigration, learning academic research methods, and preparing for the management of health systems in the future were the primary motivations of students who entered the MPH programmes [23]. In addition, MPH students in Canada and Iran agreed that an MPH programme is a good guide for them with regard to becoming competent health professionals in their future career, and that it can develop their ability to interact with various groups of people in society [24, 25]. In China, the short developmental history of the full-time MPH programme means that the teaching system is imperfect and there is a very small number of graduates, so MPH students do not understand the future employment situation. From 2009 to 2015, the number of MPH students was lower than that taking a public health academic degree [5]. Most factors influencing whether students participated in an MPH programme were related to its teaching process or to their future career.

Several previous studies have found that graduate students’ satisfaction level can reflect the gap between their studying experiences and their expectations of the programme [26, 27]. However, the existing studies conducted in China, as well as those carried out in other countries, only focused on students’ satisfaction level or factors that influenced their choices of speciality, but ignored issues such as the connections between satisfaction level and the identity of their speciality degree in the training process. The present study aimed to evaluate graduate students’ level of satisfaction with full-time MPH education in China, and whether they would again apply for this programme if they had another chance to do so. In addition, we also aimed to identify the factors that influence students’ decision-making processes. This study is the first representative nationwide survey of full-time MPH education to be conducted in China, and may provide an in-depth analysis of associated quality issues and challenges.

## Methods

The study protocol was approved by the Chinese Society of Academic Degrees and Graduate Education (Reference 2013Y02–002), and the 3-year full-time MPH graduate programme was developed by the Chinese government in 2012. Enrolled students are required to pass designated compulsory and elective courses during the first term or first year, undertake an internship or practical training in the following 6 months or 1 year, and then succeed in defending their dissertation in order to obtain their master’s degree.

### Participants

All full-time students enrolled between 2012 and 2014 in any of the 44 universities or colleges providing the MPH programme in China were eligible. After an 80-day online recruitment procedure between April and June 2015, 702 MPH students from 35 (79.5%) of these 44 universities or colleges voluntarily completed the online questionnaire. Measures were taken to guarantee that only full-time MPH students participated, and each participant could only provide information once.

### Instruments

Our questionnaire was developed on the basis of those used in previous relevant studies [28–32] and the annual Chinese graduate student satisfaction survey [12, 33, 34], and was tested using five MPH students from Peking University. An online version of the questionnaire based on Sojump, which is a tool for network research, was used for data collection. The first part of the questionnaire related to respondent characteristics, including university attended, gender, age, grade, status prior to admission and their initial willingness when they had applied for graduate study. The remaining 30 questions concerned level of satisfaction with the entire graduate educational experience, including admission, goals-setting, theoretical courses, practical training, research activities and mentorship, and were evaluated using the five-point Likert scale, ranging from five for ‘very satisfied’ to one for ‘very dissatisfied’ for each item.

### Statistical analysis

Statistical Package for Social Sciences software (Version 20.0, SPSS Inc., Chicago, IL, USA) was used for statistical analysis. Exploratory factor analysis was performed to screen and categorise the factors with common and related characteristics among the 31 variables [35]. Principal component analysis and varimax with the Kaiser normalisation rotation method were used to extract common factors. Since only the 422 students who had completed an internship or had practical training experience were evaluated on level of satisfaction with their practice, the mean of the other students for which the relevant data were available was assigned for the missing data in the factor analysis. Several rounds of factor analyses were performed until the cumulative contribution rate was greater than 70%. Kaiser-Meyer-Olkin and Bartlett’s tests were employed to ensure completion of factor analysis (KMO = 0.961 > 0.8, p < 0.001). We then generated a distribution of satisfaction scores for each aspect of the MPH programme, using the mean and standard deviation (SD). The Student’s *t*-test was used to identify any statistically significant difference between the means of the two groups, that is, those who answered ‘yes’ (group one) or ‘no’ (group two) to the question of whether they would again choose to study MPH as a graduate degree if given the opportunity. Finally, the data were analysed in consideration of variables covering six categories to identify independent factors influencing satisfaction level, using an unconditional logistic regression model.

## Results

### Demographic information

A total of 207 (29.49%) male and 495 (70.51%) female full-time MPH students studying at either ‘985’ (206, 29.34%) or ‘non-985’ (496, 70.66%) universities in 2012 or before (145, 20.66%), 2013 (246, 35.04%) and 2014 (311, 44.30%) were recruited to our online survey. Colleges and universities within the ‘985 Programme’, also known as ‘the world class university’ project, are representative of Chinese higher education development. They enjoy greater investment and a higher standard of teaching, and generally have a good reputation. The name comes from a speech delivered by President Jiang Zemin at Peking University’s centennial ceremony on 4 May 1998.

The students’ median age at admission to the MPH programme was 25 years, with a range from 21 to 46 years, showing a skewed distribution. Before admission to the programme, the majority of the participants (575, 81.91%) were new graduates, while the remainder were either employed (91,12.96%) or had not been employed (36, 5.13%) following graduation from their undergraduate degree. On application for a Master’s degree, the majority of our participants’ first preference was for the Master of Science degree (412, 58.69%), while only 31.91% (224) opted for the full-time MPH as their first choice (Table 1).

**Table 1:** Characteristics of the 702 students in the full-time Master of Public Health programme

Characteristic	Number	Percent
Type of universities providing Master of Public Health (MPH) programme		
985^a^	206	29.34
Non-985	496	70.66
Gender		
Male	207	29.49
Female	495	70.51
Age group		
21–25 years	399	56.84
26–30 years	282	40.17
30+ years	20	2.85
Unknown	1	0.14
Year of admission to MPH programme		
2012 or before	145	20.66
2013	246	35.04
2014	311	44.30
Employment status before admission to MPH programme		
New graduates	575	81.91
Employed graduates	91	12.96
Graduates who had never been employed	36	5.13
Master’s programme preference on application		
Full-time MPH	224	31.91
Master of Science	412	58.69
Other specialities	66	9.40

^a^985 Programme, also known as ‘the world class university’ project in China. Colleges and universities within the 985 Programme are representative of Chinese higher education development. They generally have a good reputation, and also enjoy greater investment and a higher standard of teaching. The name comes from a speech on 4 May 1998, delivered by President Jiang Zemin at Peking University’s centennial ceremony

### Students’ willingness

**Fig. 1:**
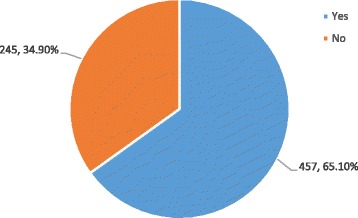
Students’ responses to the question ‘If you have another chance, would you apply for Master of Public Health as your graduate degree programme?’

Figure 1 shows the students’ responses to the question ‘If you had another chance, would you like to apply for MPH as a graduate degree?’ Over half of the students (457, 65.10%) answered ‘Yes’, and 34.90% students answered ‘No’, meaning that more than half of the students were satisfied with the MPH programme.

### Factor analysis

As a result of the principle component analysis, four common factors with a cumulative contribution rate of 70.44% were extracted from the 21 questions from our online survey that relate to features of the MPH programme, on the basis of the scree plot and parallel analytical strategy [36].

Specifically, 12 questions were categorised under common factor 1, ‘admissions & lecture courses’; nine questions under common factor 2, ‘research activities & mentorship’; seven questions under common factor 3, ‘practical training’; and three questions under common factor 4, ‘training goals’. Common factor 1 explained 28.30% of the total variance, while common factors 2, 3, and 4 explained 17.97, 17.52 and 6.65% of the total variance, respectively. The corresponding Cronbach alpha coefficients were 0.954, 0.940, 0.911 and 0.857 for the four common factors, respectively, showing high internal reliability. The pattern and structures of the rotated common factors are shown in Table 2.

**Table 2:** Results of the factor analysis of 702 Master of Public Health students

Factors	Common factors				Communality variance
	1	2	3	4	
Admissions	0.432				0.400
Course goals	0.784				0.762
Rationality of course schedule	0.768				0.706
Advancement of course contents	0.778				0.750
Professionalism of course contents	0.825				0.776
Comprehensiveness of course contents	0.772				0.698
Diversity of teaching methods	0.779				0.700
Rationality of course evaluation system	0.689				0.621
Course can meet student needs	0.798				0.781
Connection between course and practical training	0.796				0.754
Connection between course and research activities	0.674				0.633
Graduation thesis with practice	0.589	0.544			0.713
Goals can be effectively reached through research activities	0.549	0.576			0.729
Research activities can help with regard to the students’ jobs in the future	0.509	0.559			0.638
Evaluation of graduation thesis	0.524	0.541			0.648
In-campus mentors’ guidance		0.792			0.731
Occupational-orientation		0.807			0.780
Interaction between mentors and students		0.816			0.805
Off-campus mentors’ guidance		0.810			0.758
Joint guidance of on- and off-campus mentors on students’ graduation theses		0.756			0.725
Practical training goals			0.794		0.743
Conditions of practical bases			0.809		0.737
Internship subsidy			0.627		0.469
Selected practical bases			0.785		0.668
Goals can be reached through practical training			0.831		0.777
Practice can meet student needs			0.818		0.749
Practice can help with graduation thesis			0.786		0.695
Goals can guide lecture courses	0.480			0.646	0.751
Goals can guide practical training	0.535			0.554	0.712
Goals can guide research activities				0.687	0.722
Eigenvalue	8.49	5.39	5.26	1.99	
Contribution variance (%)	28.30	17.97	17.52	6.65	

### Satisfaction with the MPH programme

The participants who would choose MPH as their graduate degree if they had another chance to make the decision showed statistically significantly higher satisfaction scores with regard to admissions & lecture courses, research activities & mentorship, practical training and training goals in general, as well as on each compositional item in these four categories, compared to those who would not make this choice (student’s *t*-test, *d* > 0.2, *p* < 0.05). Among all four common factors, common factor 2 ‘research activities & mentorship’ was associated with the highest satisfaction scores, as rated by all participants (mean = 3.84) and the subgroup who would again choose the MPH programme (mean = 3.98), while the corresponding satisfaction score was only 0.02 point different from the highest reported by those who would not choose to take the MPH degree again. The lowest satisfaction scores were associated with the category of ‘training goals’ for all participants (mean = 3.52) and the corresponding two subgroups who either would or would not choose the MPH programme again (means = 3.68 and 3.21, respectively).

In the category of admissions & lecture courses, satisfaction scores ranged from 3.41 for ‘advancement of course content’ to 3.71 for ‘rationality of course evaluation system’. Correspondingly, with regard to the mean satisfaction scores in the category of research activities & mentorship, ‘research activities can help with regard to the students’ jobs in the future’ was associated with the lowest (mean = 3.67) score, ‘in-campus mentors’ guidance’ received the highest (mean = 4.1) score and ‘off-campus mentors’ guidance’ was associated with the second highest (mean = 3.99) score. For common factor 2, practical training, ‘Internship subsidy’ received the lowest (mean = 3.28) satisfaction score, while the highest score was given to ‘conditions of practical bases’ (mean = 3.89). In terms of the category of training goals, the students showed the highest level of satisfaction with ‘the goals could guide research activities’ (mean = 3.65), while ‘guide practical training’ was reported as being the least satisfactory (mean = 3.38) (Table 3).

**Table 3:** Relationship between Master of Public Health (MPH) students’ satisfaction scores on different aspects of the MPH programme and responses to the question of whether they would apply again for the MPH degree, given the opportunity (*n* = 702)

Satisfaction	Total	Yes		No		Cohen’s *d*	*p*-value*
	mean	mean	SD	mean	SD		
Component 1-admission & lecture courses	**3.53**	**3.81**	**0.51**	**3.61**	**0.54**	**0.38**	**<0.001**
Admissions	3.44	3.88	0.76	3.49	0.93	0.46	<0.001
Lecture courses							
Course goals	3.55	3.69	0.80	3.28	0.86	0.50	<0.001
Rationality of course schedule	3.52	3.60	0.92	3.23	0.88	0.41	<0.001
Advancement of course contents	3.41	3.66	0.81	3.24	0.85	0.51	<0.001
Professionalism of course contents	3.50	3.56	0.90	3.14	0.86	0.48	<0.001
Comprehensiveness of course contents	3.47	3.64	0.83	3.24	0.81	0.49	<0.001
Diversity of teaching methods	3.52	3.65	0.89	3.28	0.83	0.43	<0.001
Rationality of course evaluation system	3.71	3.85	0.75	3.44	0.84	0.52	<0.001
Courses can meet student needs	3.51	3.65	0.83	3.26	0.84	0.47	<0.001
Connection between course and practical training	3.42	3.56	0.93	3.14	0.91	0.46	<0.001
Connection between course and research activities	3.62	3.76	0.78	3.36	0.87	0.48	<0.001
Graduation thesis with practice	3.70	3.86	0.80	3.40	0.93	0.53	<0.001
Component 2-Research activities & mentorship	**3.84**	**3.98**	**0.66**	**3.59**	**0.74**	**0.56**	**<0.001**
Research activities							
Goals can be effectively reached through research activities	3.75	3.88	0.76	3.50	0.89	0.46	<0.001
Research activities can help with regard to the students’ jobs in the future	3.67	3.85	0.83	3.34	0.93	0.59	<0.001
Evaluation of graduation theses	3.82	3.94	0.70	3.59	0.79	0.47	<0.001
Mentorship							
In-campus mentors’ guidance	4.10	4.21	0.70	3.89	0.91	0.39	<0.001
Occupational-orientation	3.88	4.02	0.80	3.63	1.00	0.43	<0.001
Interaction between mentors and students	3.90	4.09	0.78	3.81	0.94	0.32	<0.001
Off-campus mentors’ guidance	3.99	4.00	0.74	3.79	0.84	0.27	0.013
Joint guidance of on- and off-campus mentors on the students’ graduation theses	3.76	3.91	0.89	3.49	1.02	0.44	<0.001
Component 3-Practical training (*n* = 422)^a^	**3.74**	**3.81**	**0.51**	**3.61**	**0.54**	**0.38**	**<0.001**
Practical training goals	3.85	3.96	0.70	3.63	0.78	0.45	<0.001
Conditions of practical bases	3.89	3.97	0.68	3.73	0.80	0.32	0.002
Internship subsidy	3.28	3.43	1.08	2.99	1.18	0.39	<0.001
Selected practical bases	3.86	3.95	0.72	3.69	0.84	0.33	0.002
Goals can be effectively reached through practical training	3.83	3.93	0.73	3.65	0.80	0.37	0.001
Practice can meet student needs	3.75	3.87	0.78	3.51	0.90	0.43	<0.001
Practice can help with graduation thesis	3.74	3.88	0.76	3.49	0.93	0.46	<0.001
Component 4-Training goals	**3.52**	**3.68**	**0.74**	**3.21**	**0.74**	**0.64**	**<0.001**
Goals can guide lecture courses	3.53	3.69	0.79	3.24	0.87	0.54	<0.001
Goals can guide practical training	3.38	3.56	0.91	3.04	0.87	0.58	<0.001
Goals can guide research activities	3.65	3.81	0.79	3.34	0.89	0.56	<0.001

^a^Only 422 students had been involved in the practical training at the time of the investigation

^*^Student’s t-test analysis of independence was performed to test differences between satisfaction scores of respondents in the responses to the question of whether they would apply again for the MPH degree

The bold is to highlight 4 factors

### Influencing factors on students’ willingness

The level of satisfaction with the current MPH programme, the type of university attended and the year of admission, as well as the initial graduate programme that had been followed, significantly and independently influenced the participants’ decisions as to whether they would again choose to apply for the MPH programme if they had another opportunity to do so. Participants who were satisfied with their current MPH programme, including performance on admissions & lecture courses, research activities & mentorship, practical training, and training goals, and those who had chosen the MPH programme as their current graduate degree programme, were more likely to choose the former again if they had another opportunity. The relevant point estimates for odds ratios ranged from 1.24 to 2.87. In addition, those participants from 985 universities and those who had been recruited to the MPH programme the earliest were less likely to choose it again as their postgraduate programme if they were provided with another chance to make the decision. The corresponding point estimates for odds ratios ranged from 0.51 to 0.58. Of all the considered influencing factors, the initial major preference showed the highest odds ratio (Table 4).

**Table 4:** The results of logistic regression in terms of students’ willingness to again choose Master of Public Health as a postgraduate degree

Variables	β-coefficient	Standard error	*χ* ^2^	OR	*P*-value
Satisfaction					
Admissions & lecture courses	0.47	0.09	25.36	1.59	<0.001
Research activities & mentorship	0.38	0.09	18.33	1.46	<0.001
Practical training	0.21	0.09	5.37	1.24	0.02
Training goals	0.46	0.09	25.63	1.59	<0.001
University type					
985 versus non-985	−0.67	0.19	12.67	0.51	<0.001
Gender					
Female versus male	0.22	0.19	1.24	1.24	0.27
Age group					
21–26 years versus 30–46 years	0.69	0.64	1.84	2.00	0.28
26–30 years versus 30–46 years	0.62	0.10	1.04	1.86	0.31
Time since admission					
2012 or before 2012 versus 2014	−0.58	0.27	4.53	0.56	0.03
2013 versus 2014	−0.55	0.22	6.48	0.58	0.01
Status before admission					
New graduates versus previous students who have never had a job	−0.54	0.43	1.57	0.59	0.21
Previous students who have had a job versus previous students who have never had a job	−0.09	0.51	0.03	0.91	0.86
Initial willingness in graduate degree application					
Full-time Master of Public Health versus other specialities	1.06	0.33	23.65	2.87	0.001
Academic degree versus other specialities	0.07	0.30	10.31	1.08	0.81

## Discussion

In applying for a graduate programme, a minority of our participants opted for the full-time MPH programme as their first choice. Six of every ten participants preferred a graduate programme that led to the academic degree of public health, or other specialities. This lack of popularity of the MPH programme may have been due to its limited familiarity among the public, since it was developed in China less than 10 years ago.

According to previous studies, the MPH programme, a recently developed professional degree programme, has not been widely recognised by the Chinese public health industry, meaning that many students had concerns with regard to their level of competitiveness in the employment market [6, 7, 37]. However, in published satisfaction studies, one national postgraduate survey showed that 71.7% of the participants were satisfied with their graduate programme [38], with the mean satisfaction score among full-time MPH students being 3.93. In another study, involving 20 students majoring in professional degrees and 1,465 student participants from nine universities, 62.1% of the professional-degree students reported higher satisfaction scores than students in other graduate degree programmes [39]. In the current study, the mean satisfaction scores were greater than three, indicating a high satisfaction level and showing that, through academic training, the students had recognised the advantages of the MPH programme after they had become more familiar with it. Therefore, our results support the previous finding [6] that dissemination of a full-time MPH programme, such as introduction of the contents and an overview of career options following graduation, are necessary to attract more students to apply for, and enroll in, such a programme.

In evaluating the level of satisfaction with the MPH programme, mentorship was associated with the highest level, while the participants were least satisfied with the double-tutor system (on- and off-campus mentors), irrespective of their answers to the question of whether they would again choose the MPH programme. The double-tutor system, designed to be a distinguishing feature that makes the MPH programme different from other degree programmes, means that students have two mentors: one from their university and the other from the Chinese Center for Disease Control and Prevention or another public health agency. Most students chose their on-campus mentor’s research project as their graduate thesis, and few preferred to do research work during an internship. Previously published studies have shown that challenges such as how to maintain the confidentiality of the project and how to address problems of authorship resulted in difficulties with regard to the two mentors joint guidance system. It would be interesting to further explore the mechanism of the double-tutor system in future studies [40–42].

Practical training is essential to the success of the entire MPH programme, as it may allow students to gain experience of working in public health agencies and the skills necessary to do so. In our survey, the participants had a higher level of satisfaction with variables such as ‘practice goals’ and ‘condition of bases’, while they were least satisfied with ‘internship subsidy’. An internship subsidy is not mandatory, so it is not provided by all training organisations and universities, which may reduce students’ enthusiasm towards participating in training, and consequently influence the success of their internship. An increase in the financial support available for professional degree programmes is a government responsibility that could help to improve the quality of the MPH programme.

The present study showed that, compared to the other three aspects, items involving courses, especially course contents and their impracticality with regard to real work, were evaluated as being the least satisfactory. Since the first year is crucial to students’ performance in subsequent practice and research activities, improvement of course contents and teaching methods is another urgent task in ensuring the success of MPH education.

Our study also showed that satisfaction with the MPH programme was significantly positively associated with the participants’ willingness to choose this programme if again they were given another opportunity to do so. The higher the satisfaction level, the more they were willing to choose MPH education. These results explained why many students had changed their attitude towards the MPH programme after admission. It was suggested that, as a whole, this programme was a success. Students are able to acquire knowledge and gain abilities to equip them for their future career. Our findings favour MPH education, and, without doubt, a greater number of undergraduate students should be made aware of the success and advantages that are associated with MPH when they choose it as their first preference. In addition, the ranking of the universities attended by the participants and participant age at admission also exerted an influence on their choice. Our findings were consistent with published studies showing that student attitude plays an essential role when applying for a graduate programme. Our results, along with the previously reported findings, are helpful for further improvement of the MPH programme [27].

The current study has at least three potential limitations. Firstly, our data could not explain why students reported lower satisfaction for the courses provided than other aspects. It is expected that this limitation will be addressed in future studies. Secondly, no students from nine universities provided any information for the current study, which may restrict the generalisation of our results. Last, but not least, some important external environmental factors that could influence students’ attitudes towards the MPH programme, beyond factors considered in the current study, such as macro policies or learning and living conditions, were not included.

## Conclusion

A total of 702 full-time MPH students from 35 universities and colleges showed a high level of satisfaction with most aspects of their MPH education, although some improvements and reforms are required. Our participants also gave positive responses to the question of whether they would choose MPH again as their graduate degree if they were given another opportunity to do so.

## Abbreviation

MPH: Master of public health
